# Thermoregulatory responses of elite ski mountaineers during simulated races under cold, hypoxic conditions

**DOI:** 10.3389/fphys.2026.1831717

**Published:** 2026-07-08

**Authors:** Tobias Dünnwald, Wolfgang Schobersberger, Anika Köck, Yannis Pitsiladis, Panagiotis Verdoukas, Hanns-Christian Gunga, Sebastien Racinais

**Affiliations:** 1 Institute for Sports Medicine, Alpine Medicine & Health Tourism (ISAG), Private University for Health Sciences and Health Technology, UMIT Tirol, Hall in Tirol, Austria; 2 Tirol Kliniken GmbH Innsbruck, Innsbruck, Austria; 3 Department of Biology, Hong Kong Baptist University, Hong Kong, Hong Kong SAR, China; 4 Centre for Exercise Science and Medicine (CESAME), Hong Kong Baptist University, Hong Kong, Hong Kong SAR, China; 5 Human Telemetrics LTD, London, United Kingdom; 6 Institute of Physiology, Center for Space Medicine and Extreme Environments Berlin, Charité –Universitätsmedizin Berlin, Berlin, Germany; 7 CREPS Montpellier - Font-Romeu, Environmental Stress Unit, Montpellier, France; 8 DMEM, University of Montpellier, INRAE, Montpellier, France

**Keywords:** core temperature, hypoxia, Olympic discipline, ski mountaineering, skin temperature

## Abstract

**Background:**

During Ski mountaineering (SkiMo), athletes are exposed to multiple environmental stressors, notably cold temperatures and reduced oxygen availability. However, how athletes thermoregulate under such conditions remains unknown.

**Methods:**

This observational study investigated the thermal response of elite SkiMo athletes exercising under cold/windy conditions at altitude. Core (T_core_) and skin (T_skin_) temperature were continuously recorded in 11 elite and 2 national SkiMo athletes (10 males, 3 females) during simulated races performed at an altitude of 2600m above sea level using ingestible electronic pills and T_skin_ sensors placed on the chest, arm, thigh, shin and hand, respectively. Each athlete completed four 15-minute uphill skiing runs at 85-90% HR_max_, each separated by a 2-3-minute downhill ski back to the starting position. Heart rate and speed were continuously recorded. Ambient temperature ranged from -8.3 °C to 1.3 °C, with wind chill temperatures reaching -11 °C.

**Results:**

T_core_ significantly increased from baseline (37.4 ± 0.2 °C) to warm-up (38.2 ± 0.2 °C, p ≤ 0.001). Thereafter, 46% of athletes maintained their T_core_ (+0.2 ± 0.3 °C, p=0.225), with a peak of 39.4 °C in one athlete, while the remaining 54% showed a decrease of 0.8 ± 0.5 °C (p=0.003; minimum 36.5 °C). T_skin_ largely decreased on all locations (all p<0.001), reaching lowest mean temperatures on the hand (21.2 ± 2.5 °C) and thigh (22.3 ± 2.7 °C). Changes in T_core_ and T_skin_ were not related to uphill speed.

**Conclusion:**

SkiMo performed under cold and hypoxic conditions clearly lowers T_skin_ of elite athletes, especially at the extremities. Contrarily, we observed a high inter-individual variability in T_core_ responses, showing that half of athletes were unable to maintain their T_core_ despite high exercise intensities, whereas the other half increased their temperatures. The observation that some athletes increased whereas others decreased their T_core_ suggests differences in net heat balance, with some having a positive heat balance (heat gain) and others a negative heat balance (heat loss).

## 1 Introduction

Ski mountaineering (SkiMo) debuted as an Olympic discipline in the Winter Olympic Games in Milano-Cortina 2026, Italy, marking a significant milestone for the sport’s recognition and global exposure. This inclusion in the Olympic program and growing interest in this discipline further highlights the need to understand the unique physiological demands placed on athletes who engage in this winter sport.

SkiMo competitions involve various formats, mainly differing in total ascent, race duration (i.e., individual, sprint, vertical (uphill only)) and the number of athletes partaking in a race (single or team) (Bortolan et al., 2021). It is one of the most demanding endurance sports (Praz et al., 2014; Duc et al., 2011; Faiss et al., 2014), inducing high cardiopulmonary strain (Schenk et al., 2011). Comparably to cross-country skiing (Holmberg, 2015), muscles of the trunk and upper body are involved to generate propulsive forces, but much longer and steeper vertical sections are involved. During individual races, which are characterized by periods of 1.5–2 hours including several ascents (up to 1900 m of total ascent, >80% of entire course length) interspersed by short downhills (Bortolan et al., 2021), a high proportion of time is spent at intensities ranging at the respiratory compensation point (Duc et al., 2011). SkiMo is commonly performed at moderate to high altitudes, where athletes are exposed to both low ambient temperatures and reduced oxygen levels. Ambient winds (up to 70 km·h^-1^ (Praz et al., 2014) and high downhill velocities (>75 km·h^-1^) (Duc et al., 2011) further increase convective cooling, lowering the wind chill temperature (Bergeron et al., 2012). Therefore, the combination of extreme physical exertion, variable terrain and harsh environmental conditions presents unique challenges for these athletes. For example, wind chill exposure during downhill following prior sweating during ascent while being clothed with only thin and lightweight racing suits were recently proposed as preconditions to put SkiMo athletes at risk for hypothermia (Schöffl et al., 2023). Taken together, these factors highlight that, in field based SkiMo studies, it is difficult to disentangle the combined effects of cold, wind exposure, exercise intensity, clothing characteristics and altitude.

Regarding extreme weather- and altitude conditions in SkiMo, the 2024–25 competition rules of the International Ski Mountaineering Federation (ISMF), state that “[ … ] *If the Wind Chill temperature is in the -10°C to -20°C range (the moderate risk, orange in the chart) recommendations regarding cold weather protection should be made available to the athletes*” and importantly, “*If the Wind Chill temperature is colder than -20°C at any of the measurement points, the race must not start”(*ISMF, 2025*)*. In addition, to reduce the risk associated with altitude exposure, the ISMF Medical Commission instructs athletes to “*not spend more than 4 hours above 3500m, no more than 2 hours above 4000m”*, and to perform *“no racing higher than 4500m*” (ISMF, 2025). However, regarding the exposure to cold temperatures, these recommendations are not evidence based, and research is needed to give more appropriate recommendations. In addition, the ISMF requires athletes to have minimum clothing consisting of three upper−body layers (a body−hugging base layer, a long−sleeved ski suit or second layer, and a long−sleeved windbreaker jacket) and two lower−body layers (a long−legged ski suit or ski pants, and breathable windbreaker trousers). The Race Jury may require specific items to be worn or carried in the backpack (ISMF, 2025). It is currently unclear how the combined environmental stressors (i.e., cold, ambient wind, movement velocity and hypoxia) during training and competitions affect the thermal responses in these athletes and if the high metabolic heat production compensates for heat loss. Previous studies performed at moderate sub-zero temperatures at altitudes ≤735 m above sea level (a.s.l.) in alpine- (Alhammoud et al., 2021) and cross-country biathlon skiing training (Blokker et al., 2022) revealed core temperature (T_core_) to be maintained above baseline but skin temperature (T_skin_) to fall significantly, especially in the distal regions. When exercise is performed at high altitude, where athletes are concomitantly exposed to cold and hypoxia, the body must maintain oxygen delivery within vascular beds on the one hand, and control heat conservation to prevent hypothermia and cold injury on the other hand (Mugele et al., 2021), potentially leading to a different thermoregulatory response. In addition, in SkiMo competitions and trainings, the time spent at high altitudes is typically longer than during other winter-sport disciplines. Combined cold and hypoxic conditions may increase the core cooling rate, with hypoxia-induced cutaneous vasodilation being one of possible drivers for the higher heat loss (Wait et al., 2023). Laboratory studies revealed additive negative effects of cold and hypoxia on strength (Lloyd et al., 2015) and endurance exercise (Callovini et al., 2024), however, if an altered thermoregulation could be a driver for such performance impairments remains elusive. A recent mechanistic study showed that the combination of multiple external stressors such as postural and thermal stress can induce different skin blood flow responses, with legs being mostly controlled by baroreflex, whereas arm vasculature responding to both, skin temperature alterations and baroreflex activity (Fisher et al., 2024).

Therefore, the aim of this study was to define the thermoregulatory response of elite SkiMo athletes during a simulated outdoor race performed in natural high altitude and cold conditions, primarily evaluating changes in core and skin temperatures.

## 2 Materials and methods

### 2.1 Participants and study design

This observational study was performed in 13 elite/national SkiMo athletes (10 males: 27 ± 7 years, 178 ± 7 cm, 70 ± 6 kg, 21.9 ± 1.0 kg/m^2^; 3 females: 29 ± 1 years; 166 ± 5 cm, 60 ± 5 kg, 21.7 ± 1.2 kg/m^2^). Ten were elite athletes (8 males and 2 females) of the Austrian National Team of Ski Mountaineering (Tier 4) and three (2 males and 1 female) athletes belonged to the Austrian SkiMo squad (i.e., highly trained competing at a national level, Tier 3 (McKay et al., 2022)). The current study was performed during a pre-world cup competition training camp held over a period of six days at the Hintertux Glacier Ski Resort, Tyrol, Austria. Athletes were examined during a total of 17 runs (i.e., four of the 13 athletes were examined twice (two females, two males), each on two different days with a minimum period of 2 days in between. We continuously recorded core- (T_core_) and skin temperature (T_skin_). In each simulated racing session, the athlete’s heart rate and speed were recorded. Thermal sensation and perceived exertion were assessed immediately post-race.

### 2.2 Race characteristics

Simulated race was performed in late 2023 during the winter season. Athletes warmed up for 23 ± 2 minutes outdoors while moving up a ski slope. For the racing part, they performed four 15-minute uphill runs at an intensity of 85-90% HR_max_, each interspersed by a 2-3-minute downhill ski back to the start (same route as uphill). Following completion of the race, an outdoor cool-down (19 ± 4 minutes) was performed. Each uphill run started at 2600m a.s.l., with athletes reaching an average maximum altitude of 2895 ± 6m. Across the four intervals, the mean distance covered was 2867 ± 295m. All runs were performed on the same ski slope along an identical route. Across sessions, terrain/snow conditions were approximately 88% even, hard piste while 12% were uneven, softer piste. Athletes used their personal ski mountaineering clothing and equipment in compliance with the ISMF race equipment rules (ISMF, 2025). All athletes were familiarized in advance with the route/slope profile and experimental procedures. They were also instructed to perform all transitions (uphill-downhill and downhill-uphill) and the downhill segments at competition speed.

### 2.3 Environmental conditions

Ambient temperature (°C) and average wind speed (km·h^-1^) were continuously measured using a mobile weather station (WS1000 Weather Station, WeatherXM, Athens, Greece) that was placed next to the starting point. Wind chill temperature was calculated by standard formula (wind chill temperature =13.12 + (0.6215 x T) – (11.37 x V^0.16^) + (0.3965 x T x​ V^0.16^); T, ambient temperature in °C; V, wind speed in km·h^-1^)). The simulated race was performed under sunny (65%), cloudy (24%) or slightly snowy (12%) weather conditions.

### 2.4 Measurements

#### 2.4.1 Core and skin temperature measurements

T_core_ was continuously recorded (every 60 seconds, with an accuracy of 0.1 °C) using an ingestible (gastro-intestinal) electronic temperature pill (eCelsius capsule BodyCap, Caen, France). Temperature data was stored within the pill and transmitted via radio frequency to a gateway (BodyCap, Caen, France). Following the simulated race, a wired connection from a notebook to the gateway was established to download data. T_skin_ was measured using flexible thermistor surface probes (eCelsius flex, BodyCap, Caen, France; discontinued), not the ingestible capsule. Probes were affixed at five locations (i.e., chest, arm, thigh, shin and hand) using permeable, adhesive tape. Sensor adhesion was inspected post-race, any displacement was recorded, and affected data were excluded from analysis. A similar technique as for T_core_ measurements was used to download data from skin sensors. Baseline T_core_ and T_skin_ of each location were recorded during a 5-minute resting period at temperate conditions (room temperature of ∼22 °C, altitude of 2600m) following skin sensor fixation. To determine global skin temperature alterations, average skin temperature (T¯_skin_AVG_) was calculated according to Ramanathan et al (Ramanathan, 1964), using the formula 0.3∗Chest + 0.3∗Arm + 0.2∗Thigh +0.2∗Shin. For each of the four intervals (one interval consisting of one uphill and one downhill), mean T_core_ and mean T¯_skin_AVG_ was used to calculate the T_core_-to-T¯_skin_ gradient.

#### 2.4.2 Body mass, nutritional intake and sweat loss

Body mass of athletes was measured twice, i.e., immediately before the warm-up (at baseline) and after completion of the cool-down. At the same time, food and beverages were weighed and urine output was estimated by assuming 0.3L per void to calculate sweat loss by mass balance. The number of voids was recorded immediately post-race race by athlete self-report.

#### 2.4.3 Heart rate and perceptual measures

Heart rate and speed were recorded using a single-strap system per athlete: either a Polar Vantage V3 (Polar Electro, Kempele, Finland) paired with a Polar Hp chest strap, or Garmin Forerunner 35 paired with a Garmin HRM-Pro chest strap (Garmin, Olathe, KS, USA). Data were exported and processed with a standardized procedure for all devices. To determine thermal sensation and thermal comfort, a visual analogue scale ranging from blue (very cold) to red (very hot) and from white (comfortable) to black (very uncomfortable) was used, respectively. Rating was performed by letting athletes shift a horizontally moveable marker to the desired position of the color bar. Scores were shown on the back side of the visual analogue scales and visible only to the research team. In addition, perceived exertion was assessed via a 15-point BORG scale, that ranged from 6 (no exertion at all) to 20 (extremely hard) (Borg, 1982). Visual analogue scales for thermal sensation/comfort and borg scales were presented on paper cards to the athlete’s immediately post-race. No additional time points were collected.

#### 2.4.4 Clothing

During the simulated race, athletes wore their individual (national team) racing suits and a backpack. In detail, the outer layer consisted of a regular racing skin suit. As base layer, 90% of the athletes wore a net shirt or a long-sleeved/T-shirt (10%), without base pants and only underwear. Moreover, all athletes wore a beanie hat, a scarf and thin racing gloves. To simulate racing conditions, all athletes wore a backpack (similar in size and weight (300-400g)) and a SkiMo helmet. During warm-up, athletes wore additional warm-up pants and jackets (made of windproof-, GORE- TEX or PrimaLoft materials).

### 2.5 Statistical analysis

Normal distribution of data was assessed using the Shapiro-Wilk test. To determine changes in T_core_, T_skin_ of each location, T¯_skin_AVG_, HR and speed, data obtained within each uphill run (15min) and it’s consecutive downhill run (2–3 min) was averaged (resulting in 4 intervals, I1 to I4). For HR and speed, data was additionally averaged within each of the four uphill and each downhill run. A two-way repeated measures analysis of variance (ANOVA) was applied, with time (within-subjects) and sex (between-subjects), to evaluate main effects and the time × sex interaction on thermoregulatory variables (T_core_, T_skin_ (chest, arm, thigh, hand, shin) and T¯_skin_AVG_), HR- and speed. When the overall Anova was significant, Bonferroni corrected *post-hoc* tests were performed. Dependent-samples t-test were used to detect alterations in body mass, food and liquids. Between-sex differences at individual timepoints were evaluated using independent-samples t-tests. To examine differences in skin temperature between body parts (chest, arm, thigh, hand, shin), mean skin temperature was calculated as the average across measurement intervals I1–I4, and differences were analyzed using an ANOVA with Bonferroni-adjusted *post-hoc* pairwise comparisons. A sub-analysis was conducted to compare performance outcomes between participants who maintained versus those who decreased core temperature, using independent-samples t-tests. All data are reported as means ± standard deviation (M ± SD). Pearson correlation coefficient was applied to assess correlations between individual variables. The statistical significance was set at p ≤ 0.05. Data were analyzed with SPSS statistical software package for Windows (version 29.0; IBM Corporation, Armonk, NY, USA).

## 3 Results

### 3.1 Core temperature alterations

**Figure 1:**
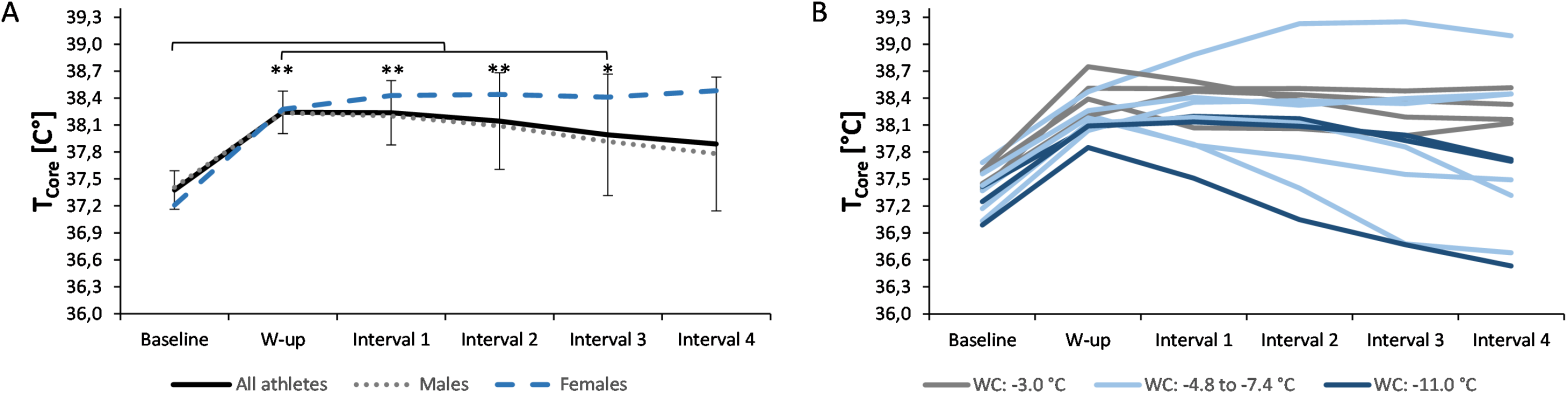
Mean **(A)** and individual changes **(B)** in core temperature (T_core_) during simulated races (n=13). Data are means ± SD. *p ≤ 0.01 and **p ≤ 0.001 for significant differences compared to baseline. ^#^p ≤ 0.05 compared to Interval 1. Significant differences refer to the main time effect, not to differences in the response between groups. Effects spanning multiple time points are indicated by a continuous capped line across the relevant interval. A p-value ≤0.05 was considered as statistically significant. WC, wind chill temperature.

**Figure 2:**
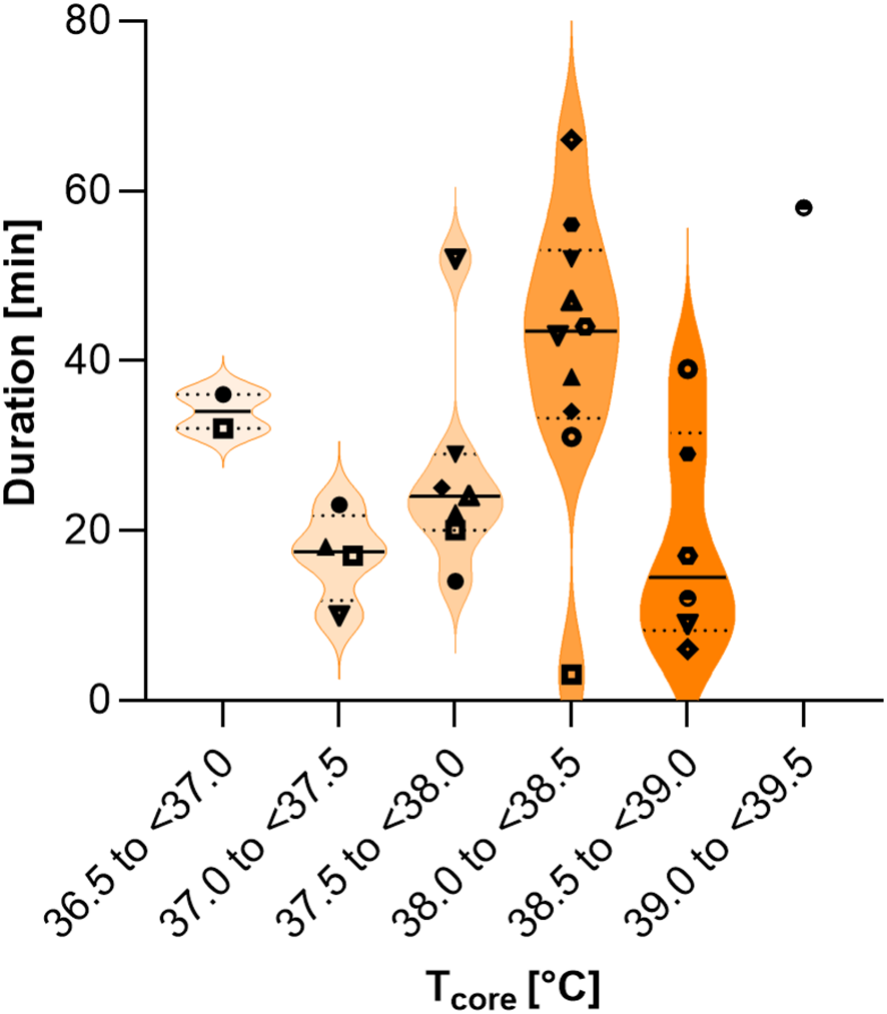
Violin plots of core temperatures (T_core_) representing the total duration spent within a specific temperature zone across all four racing intervals (n=13;11 males, 2 females), including median (bolt line) and quartiles (dotted lines). Each symbol denotes one athlete. The same symbol may appear in multiple ranges; not all athletes are present in every range.

T_core_ showed a significant time effect during the runs (p<0.001, **Figure 1A**). T_core_ increased during warm-up (38.2 ± 0.2 °C, p ≤ 0.001)) and then remained higher than baseline until I3 (38.0 ± 0.7 °C, p=0.046). In I4, T_core_ was no longer significantly different compared to baseline (37.9 ± 0.7 °C, p=0.231). No significant interaction effect for sex was observed for changes over time (p=0.152). Individual mean changes in T_core_ are presented in **Figure 1B**. In 46% of athletes, T_core_ non-significantly increased from warm-up to I4 (38.3 ± 0.2 °C to 38.5 ± 0.3 °C, p=0.225), while significantly decreasing from 38.2 ± 0.3 °C to 37.4 ± 0.6 °C (p=0.003) during that period in the other 54%. The duration athletes stayed within a specific temperature range is illustrated in **Figure 2**. Minimum and peak T_core_ reached during the runs are represented in **Table 1**. Changes in T_core_ following warm-up were not related to uphill-speed (r=0.097, p=0.777) or wind chill temperature (r=0.239, p=0.453).

**Table 1:** Mean minimum and mean peak T_core_ reached during simulated races.

Subset	Mean minimum T_core_ (absolute minimum) [°C]	Mean peak T_core_ (absolute maximum) [°C]
All athletes	37.7 ± 0.6 (36.5)	38.5 ± 0.4 (39.4)
*Males*	37.6 ± 0.7 (36.5)	38.4 ± 0.7 (39.4)
*Females*	38.1 ± 0.2 (38.0)	38.8 ± 0.2 (38.9)

Data are means ± SD. Left column shows the mean of individual minima and the absolute minimum temperature; right column shows the mean of individual maxima and the absolute maximum temperature. n=11 males and 2 females. T_core_, core temperature.

### 3.2 Skin temperature changes

**Figure 3:**
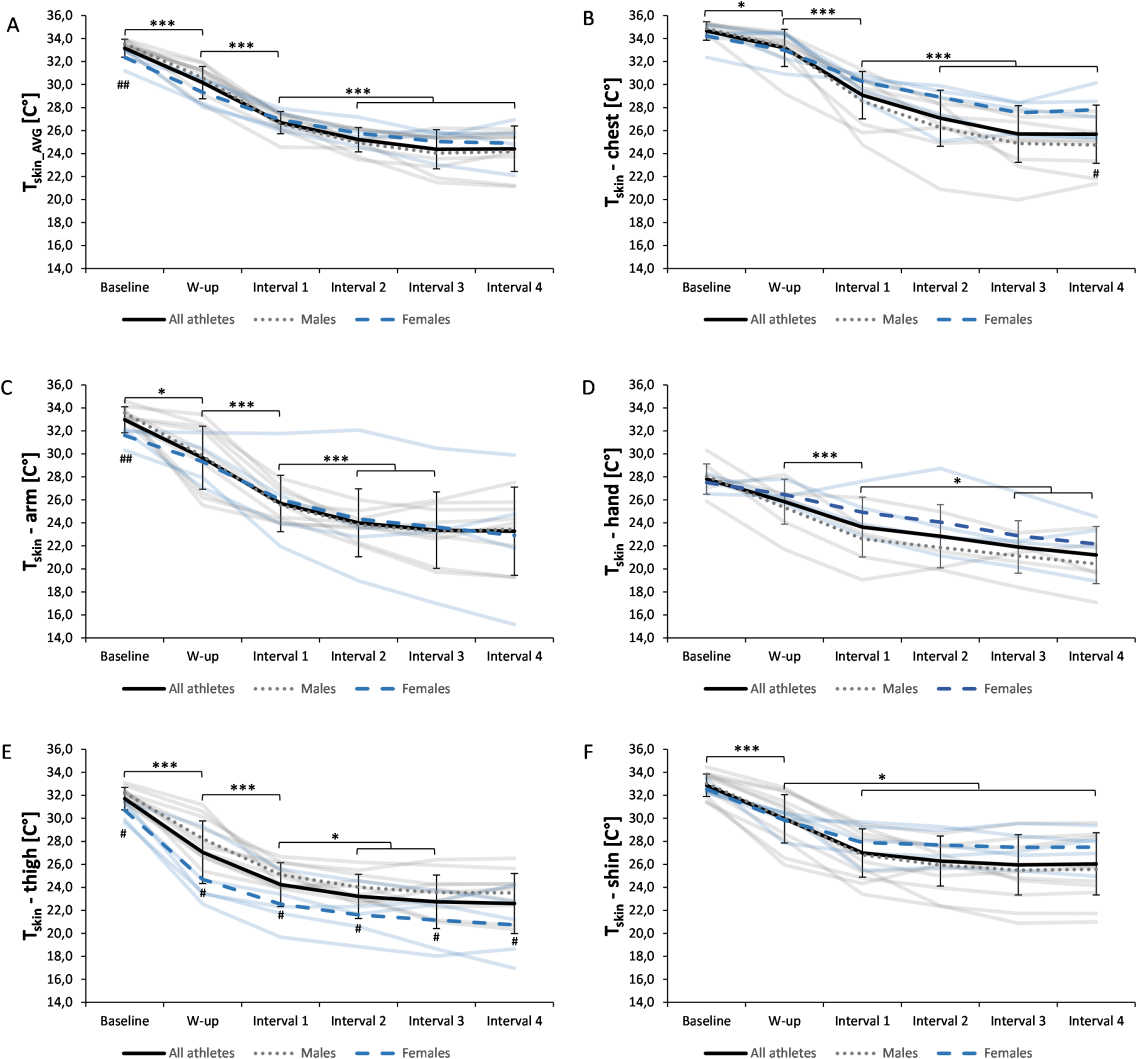
Changes of average skin temperature [T¯_skin__AVG **(A)**]; calculated according to Ramanathan) and in skin temperature (T_skin_) of the chest [n=13, **(B)**], arm [n=13, **(C)**], hand [n=9, **(D)**], thigh [n=15, **(E)**] and shin [n=15, **(F)**] during the simulated races. Data are means ± SD. *p ≤ 0.05, **p ≤ 0.01, ***p ≤ 0.001 for significant difference between two time points. ^#^p ≤ 0.05, ^##^p ≤ 0.001 for significant differences between males and females. Significant differences refer to the main time effect, not to differences in the response between groups. Effects spanning multiple time points are shown by a continuous capped line across the relevant interval. A p-value ≤0.05 was considered as statistically significant.

There was a significant time effect for T¯_skin_AVG_ during simulated races (all p<0.001) (**Figure 3A**). No significant interaction effect for sex was apparent for T¯_skin_AVG_ (p=0.127). At baseline, T¯_skin_AVG_ was significantly lower in female athletes (**Figure 3A**). T_skin_ measured on each location significantly changed over time (all p<0.001). In detail, T_skin_ of the chest (n=13), arm (n=13), thigh (n=15) and shin (n=15) significantly decreased from baseline to warm-up and tended to be lower for the hand (n=13; p=0.067) (**Figures 3B–F**). Following warm-up, a second fall in T_skin_ was observed for all five locations during I1. T_skin_ further decreased during I2-I4 on the chest, I2 and I3 on the arm and thigh as well as I3 and I4 on the hand. T_skin_ of the shin remained unchanged from I1 onwards.

No significant interaction effect for sex on T_skin_ of the chest (p=0.061), arm (p=0.509), hand (p=0.216), thigh (p=0.431) and shin (p=0.057) was observed. However, T_skin_ of the arm differed at baseline (**Figure 3C**), while T_skin_ of the thigh was significantly lower in females compared to males at each timepoint (**Figure 3E**). T_skin_ of the chest tended to be lower in males than in females during I1-I3 (all p<0.10) and was significantly lower in I4 (**Figure 3B**). In addition, T_skin_ of the shin tended to be lower in males at I2 (p<0.10). No significant sex differences were observed for the hand.

**Figure 4:**
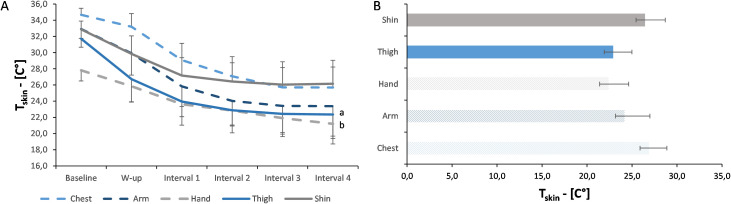
**(A)** Skin temperature by body region from baseline through exercise interval 4. Statistics were performed on the mean across exercise intervals 1-4 (chest, n=13; arm, n=13; hand, n=9; thigh, n=15; shin, n=15). Superscripts a and b both indicate a significant difference versus chest and shin (p<0.01), respectively. A p-value ≤0.05 was considered statistically significant. **(B)** Mean skin temperature by body region averaged across exercise intervals 1-4. Data are means ± SD.\.

Comparison of body parts revealed that mean T_skin_ (mean of intervals I1-I4) was significantly lower on the hand (22.4 ± 2.3 °C) and thigh (22.9 ± 2.0 °C) compared to the chest (26.9 ± 2.1 °C) and shin (26.5 ± 2.4 °C) (all p<0.01; **Figure 4**). In addition, mean T_skin_ of the arm (24.2 ± 3.1 °C) tended to be lower than temperature measured on the chest (p=0.068).

**Figure 5:**
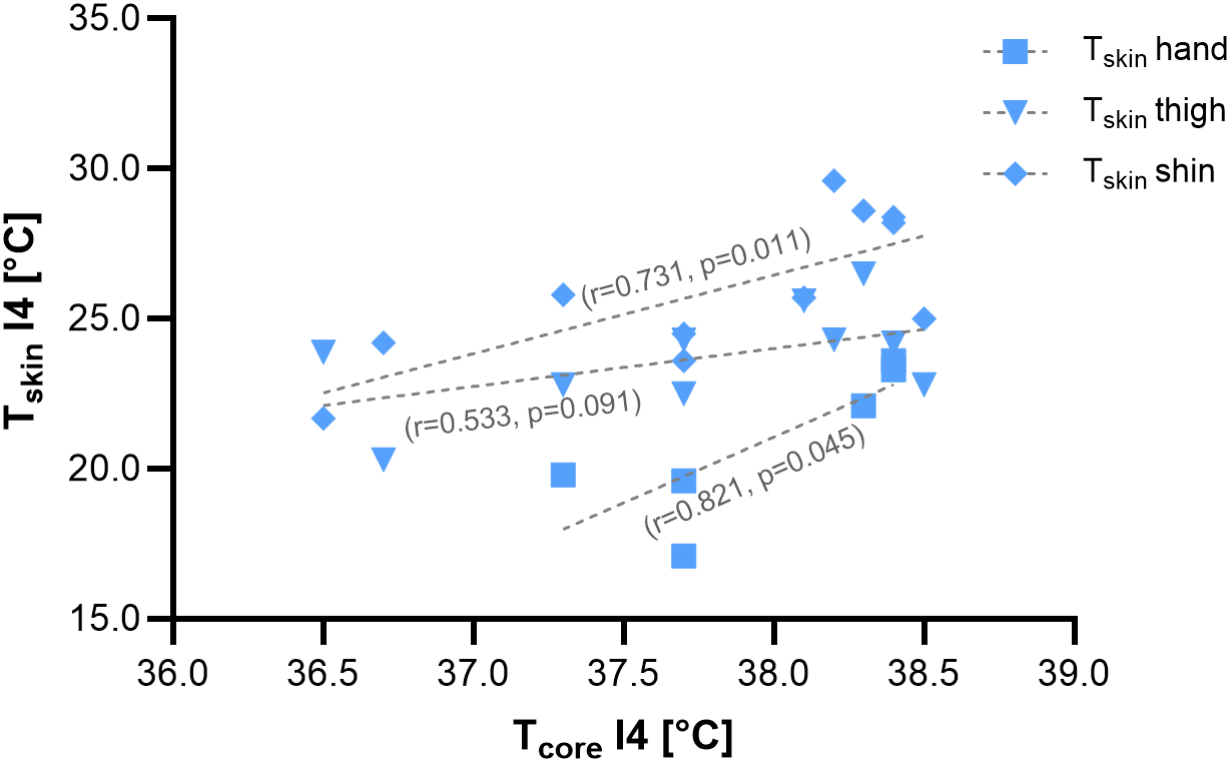
Relationship between core temperature (T_core_) and skin temperature (T_skin_) of the hand (n=6), thigh (n=11) and shin (n=11) in the final interval (I4).

The lowest temperatures measured on the five locations during uphill and downhill were 19.4 °C and 18.6 °C (chest), 14.1 °C and 14.0 °C (arm), 15.8 and 16.5 °C (hand), 16.5 °C and 15.7 °C (thigh) and 19.4 °C and 18.7 °C (shin), respectively. The T_core_-to-T¯_skin_ gradient was 11.7 ± 0.9 °C (I1), 12.9 ± 0.9 °C (I2) 13.3 ± 1.2 °C (I3) and 12.8 ± 1.3 °C (I4). T_skin_ of the hand (r=0.821, p=0.045), shin (r=0.731, p=0.011) and thigh (0.533, p=0.091) correlated with T_core_. in I4 (**Figure 5**). In addition, mean T_skin_ of the thigh (I1-I4) and T_skin_ of the thigh during downhill tended to be related to wind chill temperature (r=0.478, p=0.084 and r=0.450, p=0.093, respectively).

### 3.3 Heart rate and speed

Alterations in racing HR and speed for each interval (I1-I4) are presented in **Table 2**. HR during the uphill sections significantly changed over time. In detail, HR tended to be lower in I4 compared to I1 (p=0.083).

**Table 2:** Average changes of heart rate and speed during simulated races.

Measured variable	I 1	I 2	I 3	I 4	ANOVA *P*_time_(*η_p_²*)	
HR_uphill_ [bpm] (m+f)	168 ± 9	167 ± 9	166 ± 8	165 ± 8	.017	(.246)
HR_uphill_ [bpm] (m)	166 ± 10	165 ± 9	164 ± 9	163 ± 9		
HR_uphill_ [bpm] (f)	172 ± 4	171 ± 4	171 ± 4	169 ± 4		
Speed_uphill_ [km/h] (m+f)	1.6 ± 0.2	1.6 ± 0.2	1.6 ± 0.2	1.6 ± 0.2	.004	(.266)
Speed_uphill_ [km/h] (m)	1.7 ± 0.2^§^	1.6 ± 0.2	1.6 ± 0.2^§^	1.6 ± 0.1^§^		
Speed_uphill_ [km/h] (f)	1.5 ± 0.1	1.4 ± 0.1	1.4 ± 0.1	1.4 ± 0.1		
HR_down_ [bpm] (m+f)	140 ± 17	137 ± 16	135 ± 14	137 ± 14	.145	(.124)
HR_down_ [bpm] (m)	145 ± 12	142 ± 10	140 ± 9	142 ± 8		
HR_down_ [bpm] (f)	129 ± 23	127 ± 23	126 ± 18	127 ± 19		
Speed_down_ [km/h] (m+f)	12.0 ± 3.0	12.2 ± 2.3	11.6 ± 3.5	11.2 ± 4.2	.195	(.105)
Speed_down_ [km/h] (m)	12.6 ± 3.7	12.9 ± 2.4	12.7 ± 3.4	12.1 ± 4.7		
Speed_down_ [km/h] (f)	10.8 ± 2.5	10.9 ± 1.5	9.4 ± 2.7	9.5 ± 2.8		

Data are means ± SD. Down, downhill sections. I, interval. HR, heart rate. N, number of athletes. (m) males, (f) females (n=15; 10m, 5f). ^§^significantly different between males and females (p ≤0.05). ηp²= partial eta squared. ANOVA, analysis of variance.

A significant time effect also appeared for speed during uphill, with a tendency for slightly lower speeds in I3 and I4 compared to I1 (p=0.077 and p=0.070, respectively). There were no changes in HR (p=0.145) and skiing speed (p=0.195) during downhill sections. No significant interaction for sex appeared in uphill HR (p=0.817) and speed (p=0.151). However, uphill speed was higher in males in I1, I3 and I4 (**Table 2**). Mean uphill speed (I1-I4) and slight reductions in uphill speed from I1 to I4 did not differ between athletes that maintained or decreased their T_core_ (1.58 ± 0.06 vs. 1.62 ± 0.20 km/h, p=0.727 and -0.04 ± 0.02 vs. -0.03 ± 0.08 km/h, p=0.727; respectively).

Mean HR correlated with mean T_core_ during uphill intervals (I1-I4) (r=0.630, p=0.028) but not during downhill (r=0.253, p=0.427). Downhill speed (I1-I4) correlated with ambient temperature (r=0.767, p<0.001) and wind chill temperature (r=0.651, p=0.009), whereas uphill speed did not (r=0.377, p=0.166 and r=0.333, p=0.225, respectively). Uphill speed of I1 was inversely related to the T_core_-to-T¯_skin_ gradient (-0.792, p=0.019). No relationship was found between changes in T_core_ following warm-up and uphill speed (r=0.097, p=0.777).

### 3.4 Body mass, nutritional intake & estimated sweat loss

Body mass decreased from 67.4 ± 8.1 at baseline to 66.7 ± 7.8 kg after cool-down (0.9 ± 0.5%, p<0.001). Mean nutritional intake (i.e., beverages and food) during the sessions was 0.5 ± 0.2 kg. Urination occurred in 53% of athletes with a total of 12 voids. Mean sweat loss, calculated by mass balance (Δbody mass + intake – urine output) was 0.9 ± 0.4 L. The decrease in body mass was larger in males (-0.73 ± 0.3 kg, n=12) than females (-0.32 ± 0.4 kg, n=5, p=0.040), whereas percent body-mass change (males: -1.02 ± 0.41% versus females: -0.51 ± 0.66%, p=0.070) and sweat loss (males: −0.91 ± 0.3 L versus females: −0.74 ± 0.4 L (p=0.379) did not differ significantly between sexes. Body mass loss correlated with sweat loss (r=0.720, p=0.001).

### 3.5 Perceived exertion, subjective thermal sensation and comfort

Perceived exertion score did not depend on sex (male: 16 ± 1, females 17 ± 1, p=0.554). In addition, thermal sensation and -comfort after the simulated races did not differ between males and females (8.3 ± 4.9 vs. 8.2 ± 3.0, p=0.970 and 7.6 ± 4.8 vs. 7.7 ± 2.1, p=0.959; respectively).

## 4 Discussion

This study aimed to provide novel insights into the thermoregulatory response in elite SkiMo athletes during simulated races under cold, hypoxic conditions. With > 80% of total skiing time spent in vertical ascents, we observed heterogeneous T_core_ responses, with over half of athletes exhibiting a decline in T_core_, whereas T_skin_ decreased substantially across athletes. Alterations in T_core_ and T_skin_ were not related to speed during ascent.

### 4.1 Core temperature alterations

As expected, T_core_ increased in all athletes under daily varying environmental conditions during warm-up [+0.9 ± 0.2 °C; **Figure 1B**)]. T_core_ recorded during the first 15-minute interval was higher (38.2 °C) than that observed during a 14-minute cross-country biathlon training session (37.5 °C) performed under moderate sub-zero outdoor conditions in the field (Blokker et al., 2022). Besides a somewhat higher exercise intensity (i.e., 85-90% vs. 78% HR_max_), additional factors leading to the higher initial increases in T_core_ in our study may mainly be ascribed to SkiMo inherent characteristics such as the much steeper and longer ascents, high movement velocity only during the short downhill phase and/or additional equipment (backpack/helmet) worn by the athletes. Moreover, on most days (65%), athletes were exposed to the sun. Generally, there is a dearth of studies evaluating thermal responses in elite winter-sport athletes in the field. In alpine skiing, athletes’ T_core_ was recently reported to remain elevated (+0.3 °C) over a training period >2.5 hours at an ambient temperature of 1.9 °C (Alhammoud et al., 2021), while under laboratory conditions, T_core_ significantly declined (Suzuki et al., 2014). Both studies incorporated regular resting periods in between runs of several minutes, possibly influencing overall thermal responses to the high intensity exercise. In our study, following warm-up and after removing extra clothing, there was a large interindividual variability in the changes of T_core_ over time. As such, one male athlete reached a peak T_core_ (39.4 °C) comparable to the average peak T_core_ reached during road races of the UCI Road Cycling World Championships (i.e., 39.2 °C) performed under hot conditions (i.e., 37 °C) (Racinais et al., 2019). However, while 46% of athletes maintained or increased their T_core_ during the simulated races, the opposite trend was observed in the remaining athletes (54%; **Figure 1B**). Of those, the T_core_ of two athletes returned towards baseline levels and T_core_ of another athlete even fell below baseline by the end of their races (i.e., 36.5 °C and 36.7 °C). Comparable low T_core_ has previously been reported in professional cyclists participating in the Tour de la Provence (~4h race stage) at cool outdoor temperatures (15.6 °C, wind chill 7.8 °C), where T_core_ decreased from 37.3 ± 1.3 °C to 36.5 ± 1.4 °C (Riera et al., 2021). Notably, their findings also indicated no association between T_core_ and performance.

During the races, the decrease in T_core_ following warm-up that occurred in half of the athletes in our study does not appear to have relevantly impacted their mountaineering speed during uphill sections, as reductions in speed were not different between those who maintained or decreased their T_core_ (-0.04 vs. -0.03 km/h, respectively). Our results align with reports of maintained performance in alpine skiers during intermittent high-intensity exercise (indoor cycling) simulating ski training, in which athletes kept exercise intensities at 140% of VO_2max_, despite a decline in average T_core_ (Suzuki et al., 2014). Exercise capacity may however be impaired in case of longer or more demanding exposures, especially when a higher VO_2_ would result from lower T_core_ and T_skin_ together with a shift from fat to carbohydrate oxidation (Suzuki et al., 2014). In our study, we cannot rule out the possibility that carbohydrate intake following each downhill section may have mitigated excessive declines in blood glucose levels, thereby supporting the athletes’ ability to maintain their exercise intensity.

The high variability in T_core_ suggests the possibility that metabolic heat production may not have fully compensated for heat loss in all athletes. However, this interpretation is indirect because metabolic heat production was not directly measured. It should also be noted that heat storage cannot be inferred from T_core_ alone. Therefore, an increase or decrease in T_core_ does not necessarily indicate positive or negative heat storage because it also depends on skin and muscle temperatures. Other factors such as clothing insulation, wind exposure (airflow), solar radiation, relative exercise intensity and pacing, and body composition may also have contributed to the observed decreases in T_core_. For example, clothing was not fully standardized in our study, which likely influenced both T_skin_ and T_core_ responses. Inter-individual differences in clothing insulation and moisture accumulation may have contributed, at least in part, to the large inter-individual variability in T_core_ responses. We did not quantify clothing insulation (Clo) or garment wetness, so we are unable to apportion their effects. Nevertheless, clothing used by the athletes was similar to that worn in real competitions. The variability in T_core_ responses may also reflect individual differences in thermoregulation between athletes. Hypoxia could be a contributing factor, but this interpretation is speculative because we did not include a normoxic or low−altitude comparison or direct mechanistic measures. Although currently no definite conclusion can be drawn on the effects of hypoxia on thermal responses in the cold (Mugele et al., 2021), accelerated T_core_ cooling during rest with (Johnston et al., 1996) or without prior exercise (Arnold et al., 2021; Cipriano and Goldman, 1975) was reported in previous laboratory studies. These effects were partially explained by an attenuated vasoconstrictor- and shivering response and/or by increased respiratory heat loss (Jonston, Arnold). As such, T_skin_ was shown to decrease less at altitude (e.g., 2 °C at 5000m a.s.l.) compared to sea level (Cipriano and Goldman, 1975) and a higher cutaneous blood flow was observed (Arnold et al., 2021). However, it remains to be established how exercise as an additional stressor to cold and hypoxia acts on core and skin temperature responses.

### 4.2 Skin temperature alterations

Conversely to the changes in T_core_, alterations in T_skin_ during the simulated races were more homogeneous, showing clear decreases in T¯_skin_AVG_ and for each individual location (i.e., chest, arm, hand, thigh, shin), with most pronounced reductions observed on the hand and thigh (**Figure 3**). Similarly, in cross-country skiing training performed at ambient temperatures of -4 °C, thigh T_skin_ decreased to 22.9 °C (Blokker et al., 2022). However, the decline in T¯_skin_AVG_ and that of the thigh are considerably larger than those observed during a field-based alpine skiing training (i.e., 24.4 °C vs. 30.5 °C and 22.6 °C vs. 29.3 °C, respectively) (Alhammoud et al., 2021). The thinner clothing worn in SkiMo may be at least one underlying factor contributing to the lower T_skin_.

In our study, lower T_skin_ of the hand, shin and thigh were related to lower T_core_ in the final interval (**Figure 5**). These findings may lead to the speculation that athletes experiencing lower skin temperatures at their extremities may be more prone to lowering of T_core_ and thus to be at higher risk for an impaired thermal balance. The lower T_skin_ of the thigh was associated with lower wind chill temperatures, aligning with previous observations made under simulated cross-country skiing conditions, showing more pronounced falls in T_skin_ with lower ambient temperatures (Wiggen et al., 2016). Earlier studies suggested that a low T_skin_ may lead to decreased muscle temperature, possibly accounting for impaired exercise performance in the cold (Sandsund et al., 2012; Wiggen et al., 2016). In fact, mechanistic studies demonstrated that even mild cooling decreases muscle temperature and consequently muscular performance, and that this effect seems to be dose-dependent (Oksa et al., 1997). Changes in the neural drive (e.g., increased level of co-activation and altered agonist-antagonist ratio) were proposed to be potential underlying mechanism leading to performance degradations (Oksa et al., 2002). However, conversely to lower muscle temperature, a lower T_skin_ does not necessarily imply a decrease in performance. For example, in female athletes, a low T_skin_ together with a high T_core_ did not translate into an altered performance (i.e., time to exhaustion, speed) during time trails under cold ambient conditions (Renberg et al., 2014). In our study, we also did not observe meaningful changes in performance (i.e., speed) over the four uphill intervals. However, we cannot rule out that low T_skin_ during the simulated races influenced muscle temperature, for example by hindering further increases. Notably, we observed a higher T_core_-to-T¯_skin_ gradient during the first ascent phase to be related to a slower uphill speed. A high gradient between T_core_ and T_skin_ may be associated with higher heat loss. In their recent field study, Blokker et al. suggested that limiting skin temperature declines would be crucial, possibly illustrating the most efficient strategy to minimize the peripheral-to-core temperature gradient (Blokker et al., 2022). Therefore, when exercise is performed under cold conditions, this gradient might be an interesting parameter to look at, possibly being more sensitive in the context of exercise capacity than T_core_ and T_skin_ in isolation.

Cooling of the skin by cold exposure may also impair postural control (i.e., increase in muscular tone and postural sway) (Mäkinen et al., 2005) and dynamic balance (Montgomery et al., 2015) which, beyond impacting on performance, may increase the risk for injuries. In our study, the lowest T_skin_ were recorded during downhill sections, presumably due to the higher movement velocity. In addition, downhill speed and T_skin_ of the thigh were lower at colder wind chill temperatures. Here, due to the nature of the study design, we are unable to identify if the lower speed may be due to cold induced degradation in neuromuscular and/or sensory function. In the context of SkiMo, such a dysfunction of sensory systems (e.g., proprioception) that are involved in balance control together with a decrease in muscular strength in the lower extremities would unequivocally be relevant when skiing downhill with high skiing speeds. When athletes compete or train at altitude, the exposure to hypoxia may then illustrate an additive adverse effect on static and dynamic balance (Stadelmann et al., 2015; Muralt et al., 2025; Degache et al., 2012). However, we did not measure muscle temperature, neuromuscular performance, proprioception, postural control, or technical skiing performance in this study, so the links proposed here should be considered speculative.

## 5 Limitations

We were unable to analyze T_core_ data from all ingested pills due to technical issues during data recordings/transfer. In addition, we were not able to record skin temperature, HR and speed in all athletes (the respective number (n) of athletes is detailed in the results section and in the legends of the tables and figures).

Another limitation is that our sample size did not allow multivariable modeling to identify specific determinants of between-athlete differences in T_core_ changes. Whether a decrease in T_core_ is “good” or “bad” depends on the magnitude and duration of the decline. Nevertheless, large and sustained decreases in T_core_ increase hypothermia risk. In addition, cardiovascular- (e.g., decrease in muscle oxygenation), neuromuscular function, and metabolic regulation (e.g., shift towards greater carbohydrate use) may be impaired, potentially negatively affecting performance and health. Importantly, we did not observe a clear association between T_core_ responses and uphill performance metrics in the present cohort.

Our findings pertain to elite athletes and may not be generalized to recreational or sub-elite populations. Non-elite athletes may exhibit similar or greater variability in thermoregulatory responses due to broader differences in factors such as clothing/insulation choices, training status, acclimatization and body composition. Elite athletes may also demonstrate more consistent pacing and race strategies than recreational athletes. Future studies should compare thermal responses across different performance levels under standardized conditions to assess generalizability.

Finally, our sample size was relatively small, limiting statistical power and generalizability. In addition, our sex-related comparisons are constrained by the small number of female athletes. Therefore, our findings should be interpreted as exploratory and not overgeneralized.

## 6 Conclusion

In summary, this field-based study revealed marked reductions in T_skin_ during simulated SkiMo races in cold, high-altitude conditions with the lowest values at the hand (21.2 °C) and thigh (22.6 °C). Core temperature responses were heterogeneous. Some athletes maintained T_core_ around 38.5 °C or increased it to 39.4 °C, whereas others experienced decreases to 36.5 °C. A possible explanation is that, in some athletes, metabolic heat production did not fully offset heat loss; however, this interpretation is indirect because metabolic rate was not measured. Notably, more than half of the athletes did not maintain post–warm−up T_core_, including on days with less severe ambient conditions.

## Data Availability

The raw data supporting the conclusions of this article will be made available by the authors, without undue reservation.
